# DIEP flap salvage by cannula venesection of the superficial inferior epigastric vein

**DOI:** 10.1007/s00238-015-1177-z

**Published:** 2016-01-15

**Authors:** Puttan Wongtriratanachai, Abbe Michelle Martin, Kongkrit Chaiyasate

**Affiliations:** Division of Plastic surgery, Department of Surgery, Faculty of Medicine, Chiang Mai University, Chiang Mai, Thailand; Plastic & Reconstructive Surgery Fellow, Department of Plastic and Reconstructive Surgery, Detroit Medical Center, Detroit, MI USA; Department of Plastic Surgery, Beaumont Health System, Royal Oak, MI USA

**Keywords:** DIEP flap, Cannula, Venesection, SIEV, Venous congestion

## Abstract

The deep inferior epigastric perforator (DIEP) flap is a technique of autologous breast reconstruction that is gaining popularity. The main advantage of the DIEP flap over the traditional transverse rectus abdominis myocutaneous (TRAM) flap is that there is a lower incidence of abdominal wall donor-site morbidity with the DIEP flap. However, venous congestion is the most dreaded complication of DIEP flap surgery which requires prompt intervention. In this case report, we present a simple but effective procedure to salvage the congested DIEP flap. A 55-year-old female underwent left breast reconstruction with DIEP flap and developed venous congestion during surgery. Cannula venesection of the superficial inferior epigastric vein (SIEV) was performed intraoperatively followed by intermittent aspiration of blood for 3 days. A satisfactory aesthetic result was achieved with no evidence of fat necrosis. This procedure eliminated the need for performing an additional microvascular anastomosis, required less operative time, and allowed augmentation of the venous drainage of the congested flap.

Level of Evidence: Level V, therapeutic study.

## Introduction

The DIEP flap has proven to be a major advancement in autologous breast reconstruction. This flap has become a valuable option due to the small amount of rectus abdominis muscle fascia sacrificed causing less anterior abdominal wall morbidity such as hernia formation or bulging [1, 2]. The arterial supply of the DIEP flap is not frequently compromised, but the flap has a potential venous drainage problem. While raising the flap, it is inevitable that many perforators must be sacrificed. In conjunction with transection of the superficial veins, venous insufficiency can result.

## Case report

**Fig. 1:**
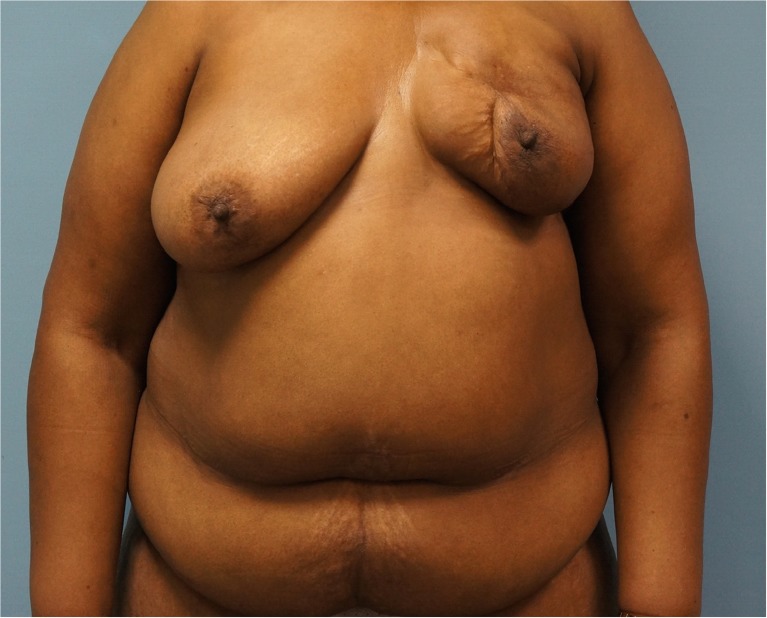
Preoperative view of a 55-year-old female patient with a history of left breast conservation surgery, radiation, and a painful scar contraction deformity

A 55-year-old female with a history of breast conservation surgery and radiation for left breast cancer presented 4 years postoperatively with a painful scar contraction deformity. She had remained cancer-free and had no other medical illness. See (Fig. 1) for the preoperative appearance of the patient. The patient’s only previous abdominal surgery was a hysterectomy, but preoperative CT angiogram of the abdomen and pelvis confirmed the presence of adequate periumbilical perforators. The surgical plan was for the general surgeon to complete a left simple mastectomy in order to fully excise the scar tissue, then the plastic surgeon would perform immediate left breast reconstruction with a DIEP flap.

After left simple mastectomy was completed, immediate reconstruction of the left breast was performed with a right-sided DIEP flap that contained three perforators. The SIEV was harvested with the flap per the surgeon’s routine. In this case, the SIEV was noted to be atypically moderately engorged immediately after division. Microvascular anastomosis was performed between the internal mammary artery and the deep inferior epigastric artery with 8-0 Nylon. Venous anastomosis was performed between the internal mammary vein and deep inferior epigastric vein with a 3.0-mm Flow Coupler (Synovis, Baxter International Inc, Alabama). Patency of the venous anastomosis without kinking was confirmed by direct inspection under the microscope, Acland (vessel strip) test, and audible flow on the implantable venous Doppler.

**Fig. 2:**
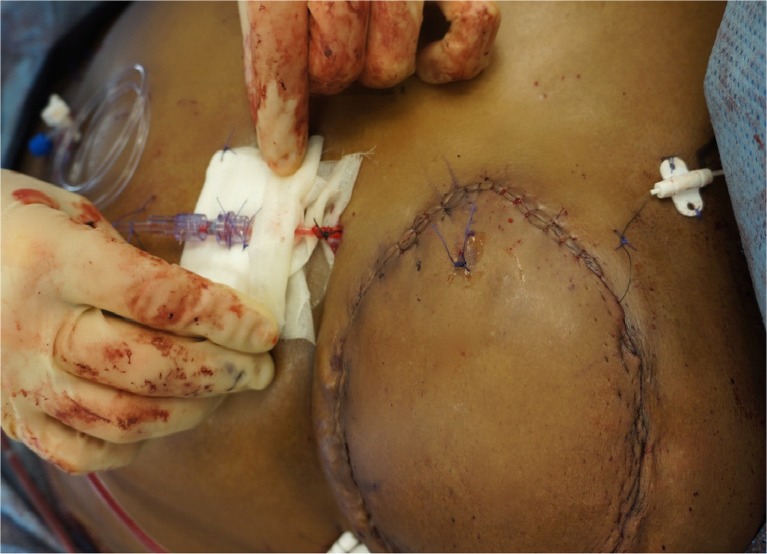
Cannula venesection of SIEV was performed intraoperatively

The flap was reperfused for approximately 20 min, and the venous doppler signal was normal. However, the flap was noted to be congested, and the clipped SIEV was engorged significantly. Inspection of the vascular pedicles revealed that there was no kinking and that the caliber of each of the anastomosed vessels was normal. The clip on the superficial vein was removed, and high-flow venous blood spurted out. In order to relieve the venous congestion and avoid fat necrosis, there were two options. The first option was to supercharge the DIEP flap by performing a retrograde anastomosis of the SIEV with a vein graft to either the internal mammary vein or the thoracoacromial vein. The second option was cannula venesection which entailed placement of a catheter into the SIEV to allow intermittent phlebotomy to relieve the pressure of the venous congestion. We chose to perform cannula venesection of the SIEV because it would require less operative time than supercharging the flap, and it would avoid having to create another microanastomosis.Venesection of the SIEV entailed inserting the cannula (Surge cardiovascular, blunt tip without check value) into the SIEV lumen, tunneling it out of the skin of the anterior chest wall at the inferomedial aspect of the reconstructed breast, and securing the cannula to the SIEV with 2-0 silk (Fig. 2). The cannula was secured to the skin with several interrupted 3-0 Prolene sutures. The three-way tap extension was kept open with heparinized saline. Venesection of the flap was performed by withdrawing 10 ml of blood every 4 h on the first postoperative day, every 6 h on the second day, and every 8 h on the third day. After 72 h of intermittent venous drainage, the cannula was withdrawn. After 72 h of augmentation of venous drainage, the flap was less congested, and the cannula was withdrawn. The total amount of aspirated blood was 110 ml. Blood transfusion was not necessary in this case.

**Fig. 3:**
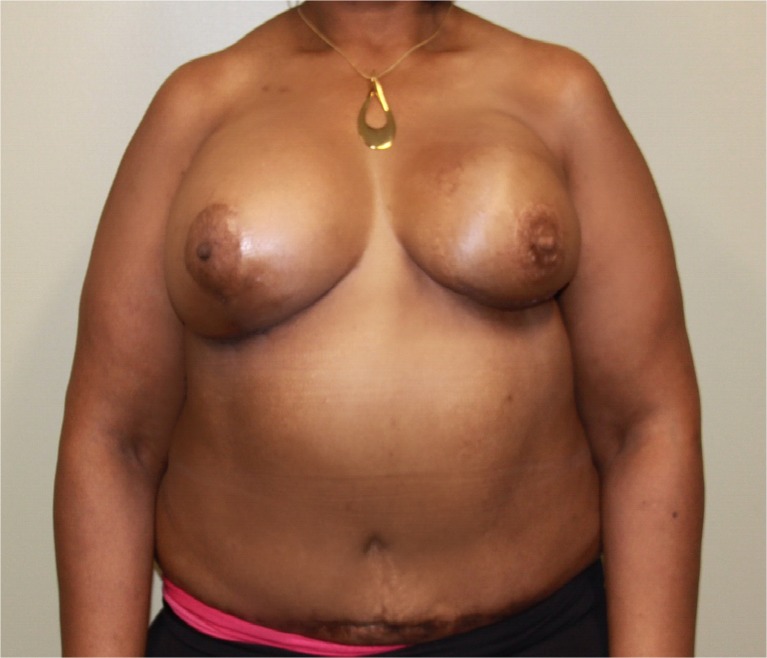
Postoperative view at 6 months after revision surgery for symmetry

The flap remained viable with no evidence of fat necrosis. The patient was discharged on postoperative day 5 and was scheduled for revision surgery for symmetry 6 months postoperatively. An acceptable aesthetic result was achieved (Fig. 3).

## Discussion

The venous anatomy of infraumbilical abdominal skin and subcutaneous tissue is variable in the DIEP flap elevation. The SIEV distributes deep branches which perforate through the anterior rectus sheath and drain into the venae comitantes of the deep inferior epigastric artery (DIEA). The small connection between this superficial and deep venous system is via the oscillating veins [3–5]. During harvest of the DIEP flap, these perforating veins are inevitably interrupted. If the remaining perforators are inadequate, flap congestion results. The incidence of venous congestion of the DIEP flap is 2–8 %, and it must be treated by venous augmentation to relieve the congestion and alleviate flap morbidity [6].

Several effective techniques to augment or supercharge the venous outflow of DIEP flap have been established [6, 7]. The reports of the SIEV to the cephalic vein, thoracodorsal vein, lateral thoracic vein, basilic vein, DIEV, thoracoacromial vein, and proximal cut end of second DIEV *venae comitantes* have been well described [8–15].

The concept of venesection of the SIEV was proposed by Hartrampf CR Jr et al., Caplin DA, and Stasch [16–18]. Hartrampf et al. inserted the arteriotomy cannula into the lumen of DIEA and DIEV and used it as a mechanical leech. Intermittent venous bleeding from the DIEV allowed the congested flap time to improve venous outflow and adapt to the new reversed blood circulation. Stasch et al. reported the success of the venesection of the SIEV. They introduced the cannula connected with a three-way tap extension into the SIEV and aspirated blood every 4–6 h.

After we noticed mild congestion of the flap and obvious engorgement of the SIEV, we decided to perform cannula venesection of the SIEV. It is a simple, quick procedure rather than the other microsurgical operative options. This procedure avoided further vessel dissection and the risk of additional microsurgical anastomosis. The total blood loss from the venesection in our case was only 110 ml, and blood transfusion was not needed.

Since the venous congestion in the DIEP flap is multifactorial, it is very important to meticulously dissect and preserve the total length of SIEV during the DIEP flap elevation. If the flap congestion is recognized, prompt augmentation of venous outflow is required. Cannula venesection of the SIEV is a safe, simple, and effective way to salvage the venous congested DIEP flap.
